# Associations of multicomponent exercise and aspects of physical performance with frailty trajectory in older adults

**DOI:** 10.1186/s12877-022-03246-6

**Published:** 2022-07-05

**Authors:** Tzu-Ying Chiu, Hsiao-Wei Yu

**Affiliations:** 1 Department of Health and Welfare, College of City Management, University of Taipei, Taipei City, Taiwan; 2 Department of Gerontological Care and Management, College of Nursing, Chang Gung University of Science and Technology, Taoyuan City, Taiwan; 3 Department of Family Medicine, Keelung Chang Gung Memorial Hospital, Keelung City, Taiwan; 4 Geriatric and Long-term Care Research Center, Chang Gung University of Science and Technology, Taoyuan City, Taiwan

**Keywords:** Frailty trajectory, Multicomponent exercise, Physical performance

## Abstract

**Background:**

Previous research has shown that frailty leads to falls, institutionalization, hospitalization, and the loss of functional capacity. While numerous intervention methods aim to reverse frailty, the most effective in older adults is multicomponent exercise. Physical performance has been highlighted as a key factor in mobility, independence, and the burden of chronic disease. Several studies have demonstrated an association between physical performance and frailty; however, the relation between the two over the long term has not yet been fully investigated. Therefore, the current study aims to examine how aspects of physical performance are associated with frailty in the long run for older adults in Taiwan.

**Methods:**

This nine-month longitudinal study employed the generalized estimating equation (GEE) modeling to identify measures associated with frailty trajectory. A sample of 159 community-dwelling older adults was recruited through purposive sampling in 12 community care centers in Taiwan. A quasi-experimental approach was adopted in which participants were assigned to the control group or to receive a multicomponent exercise intervention and examined sociodemographic, physical performance, and other factors at the baseline, post intervention (3 months), and follow up (6 months) levels. The multicomponent exercise program was designed based on the principles of the American College of Sports Medicine and comprised aerobic exercise, muscle-strengthening activities, balance training, and stretching exercises once per week for 2 h per session for 12 weeks.

**Results:**

After intervention, we found that the multicomponent exercise group exhibited better performance in the 2-minute step test than the control group (*p* < 0.05). Regarding long-term effects on frailty trajectories, the study finds that age progression, being female, and longer completion time in the timed up and go test increase the probability of frailty (*p* < 0.05). Conversely, more steps in the 2-minute step test and undertaking the multicomponent exercise program reduced the long-term probability of frailty (*p* < 0.05).

**Conclusions:**

This study is the first to explore the relation between indicators of physical performance and frailty trajectory among older adults in Taiwan. Furthermore, we provided support for the efficacy of the multicomponent exercise program in improving frailty status.

## Background

Facing the challenge of the fast-aging population, every country is devoting substantial effort to maintain health among older adults, including those who are healthy, frail, disabled, and in need of care, and is developing the framework for healthy aging [1–3]. Frailty is an important issue as people age because previous studies have demonstrated that frailty leads to fall, institutionalization, hospitalization, and the loss of function capacity [4]. In addition, numerous studies have noted that exercise is the most effective intervention for delaying or reversing frailty [5–10] regardless of how frailty is measured [9], whether by the study of osteoporotic fractures (SOF) index [11], Fried frailty phenotype [4, 12], or the frailty index [13].

Many intervention methods could reverse frailty, where exercise was the most effective one [14]; however, the types of exercise could vary worldwide. According to the recommendation of the U.S. Department of Health and Human Services [15] for maintaining health among older adults, the most suitable forms of exercise are the multicomponent ones, such as balance training along with aerobic and muscle-strengthening activities. Furthermore, extant literature has reported that multicomponent exercises can help older adults improve physical performance [8, 16–23]. Moreover, previous scholars have highlighted that physical performance is a key factor in mobility, independence, and the burden of chronic diseases [24]. Several systemic reviews have indicated an association between physical performance and frailty [23, 25], but how this relation develops in the long term has not yet been fully investigated. Against this background, it has been predicted that the program could help older adults improve their physical performance and frailty status after intervention. Therefore, the current study aims to examine associations of sociodemographic factors and measures of physical performance with frailty in the long run and the effect of the multicomponent exercise program on frailty in older adults in Taiwan.

## Methods

**Fig. 1:**
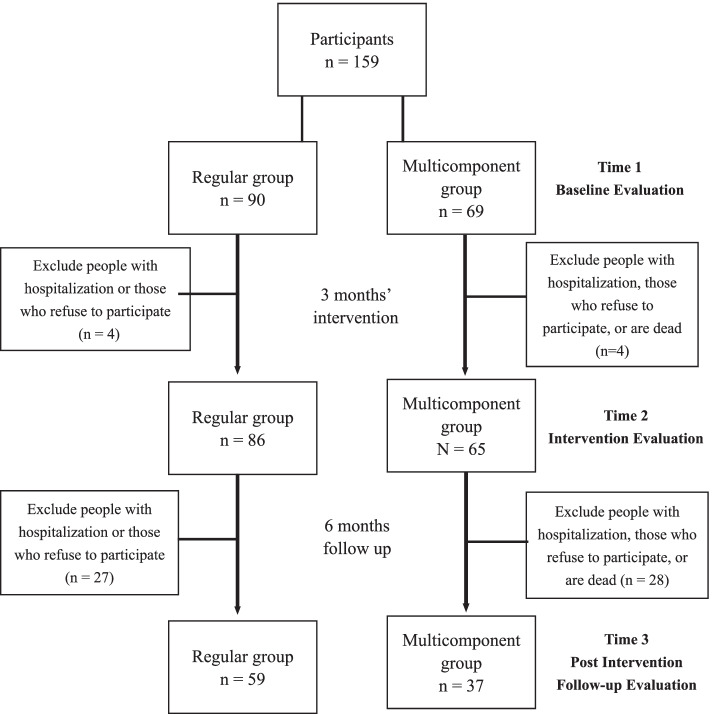
Flowchart indicating participant inclusion and exclusions

We conducted a three-wave longitudinal quasi-experimental study with face to face in 9 months. The participants were classified as a regular group and a multicomponent exercise group. The two groups underwent the questionnaire assessment for data collection over three time points from November 2018 to August 2019. We collected the baseline data before the intervention was implemented (Time 1), the intervention lasted for 3 months (Time 2), and the evaluation of multicomponent exercise maintenance was conducted after 6 months (Time 3; (Fig. 1); every evaluation was assessed by the same evaluators to maintain the test–retest validity.

### Participants

The quasi-experimental research approach was adopted. The study recruited community-dwelling older adults through purposive sampling in 12 community care centers in Taipei and Taoyuan City. Those from six community care centers were assigned to the multicomponent exercise group and those from the other six to the control group. The inclusion criteria of the six multicomponent exercise group were (1) aged > 65 years and joined government programs to prevent or delay disability in the community, (2) a score of ≥90 (mild or no disability) in the Barthel activities of daily living (ADL) index, and (3) living in the community and willing to communicate in Chinese. The exclusion criteria were (1) diagnosis of dementia and (2) recommendation from physicians to avoid joining community activities based on the health condition of older adults. The only difference between the inclusion and exclusion criteria for the regular group was older adults that did not undertake government programs to prevent or delay disability in the community; other criteria were consistence.

### Intervention

A multicomponent exercise program was designed based on the principles of the American College of Sports Medicine. The study selected median-intensity exercises for older adults, which included five sessions, namely, warm-up, aerobic exercise, muscle-strengthening activities, balance training, and stretching exercise [26]. Interventions during the first month were basic aerobic exercise and muscle-strengthening activities using a resistance band and a Swiss ball. The second month introduced advanced aerobic exercise, muscle-strengthening activities, and basic balance training. The third month was concentrated on muscle-strengthening activities and advanced balance training. Based on past reference, a multicomponent exercise program spans 3–6 months. As a session frequency ranging from once to thrice per week with a session duration of 40–120 min has shown to improve post-intervention physical performance and frailty status in older adults, the current study employed a session frequency of once per week for 12 weeks with a duration of 2 h per session. The regular group was engaged in their typical activities in the community.

### Instrument

The questionnaire comprises the following parts.

1. Demographics, such as age, gender (male/female), level of education (elementary school/junior high school/senior high school and above), numbers of diseases (sum of items, such as hypertension, diabetes, cardiac disease, hyperlipidemia, and others), falls in the last year, and exercise habits (Yes/No).
2. Assessment of physical performance using the senior fitness test (SFT).
  - The four-stage balance test evaluates the static balance and rate the ability of the participants to stand in four poses using four instructions, namely, “stand with your feet side-by-side” (Yes/No), “place the instep of one foot so it is touching the big toe of the other foot” (Yes/No), “tandem stand: place one foot in front of the other, heel touching toe” (Yes/No), and “stand on one foot” (Yes/No). The sum the four items was considered the final score (0–4).
  - Back scratch test (cm) measures flexibility in the shoulder and the distance between the hands when brought together behind the back.
  - Sit and reach flexibility (cm) demonstrates the flexibility of the extremities, such as hamstrings and the lower back.
  - Timed up and go test (seconds) illustrates the mobility and ability of individuals to maintain static and dynamic balance. The study assesses the duration of time required for the participants to walk across 2.44 m and turn back and sit down.
  - 2-minute step test (times) tests the aerobic and lower-body muscle endurance. Basically, the number of steps that participants can walk in 2 min is counted. Barring the indicator of timed up and go, other indicators exhibited high scores with improved physical fitness among older adults but not vice versa [27].
3. Functional performance: the ADL was used to measure the difficulties in conducting daily activities, such as eating, transferring from bed to chair, grooming, bathing, managing indoor mobility, going up and down stairs, dressing, toileting, and bowel and bladder incontinence [28]. The instrumental activities of daily living (IADL) was used to examine skills and interaction with the environment to fulfill daily tasks and activities (e.g., shopping); ability to handle finances, use public transportation, use telephones; housekeeping; food preparation; use of medication; and laundry [29]. Performance in each activity was assessed using a Likert-type scale ranging from 0 = no limitation to 3 = full limitation. The scores for ADL and IADL were summed, where high scores indicated worsening functional performance [30].
4. Mini-mental state examination (MMSE) was used to determine the cognitive function of the participants, such as orientation, attention, memory, language, and visual–spatial skills. Ten questions with dichotomous responses (true or false) were presented. The false items were summed and classified into four categories, namely, 0 = no cognitive impairment (0–1 false items), 1 = mild impairment (2–3 false items), 2 = moderate impairment (4–6 false items), and 3 = severe impairments (7–10 false items) [31].
5. The Kihon checklist is a 25-item self-report questionnaire on physical activity, nutrition, oral function, house-boundedness, cognitive status, and depression and uses dichotomous responses (Yes or No). Scores may range from 0 to 25 with high scores indicating high levels of need of care [32].
6. Quality of life (QOL) was examined using the EuroQol instrument (EQ-5D), which is composed of five items, namely, mobility, self-care, daily activities, pain/discomfort, and anxiety/depression. Each item was rated using three responses (1 = no problems; 2 = a few problems; and 3 = totally problems); higher score indicated worse QOL [33].
7. Frailty was assessed using the Fried frailty phenotype, which is composed of the following items.
  - Weight loss: lose 5% of the current weight compared with that of the previous year (Yes/No).
  - Low levels of endurance and energy, that is, feeling exhausted or without energy in the previous week (Yes/No).
  - Low levels of physical activity: the calorie consumption of the males was less than 383 cal per week and 270 cal per week for the female ones.
  - Weakness: evaluation of the grip strength (kg) of the participants; the cut-point standards for the male and female respondents were less than 26 and 18 kg, respectively.
  - Slowness: the time (seconds) taken for the participants to walk 15 ft.; the cut-off point for walking speed was less than 0.8 m/s. If the participants meet the three criteria, they were considered frail, whereas meeting one or two criteria indicated prefrailty [4, 12].

We applied the generalized estimating equation (GEE) model to identify possible factors correlated with the outcome variable, frailty [34]. The GEE is a robust means of analyzing longitudinal data, as it can produce regression estimates for repeated measures of non-normally distributed response variables, such as the outcome variable, frailty, in the current study. Moreover, GEE allows both time-varying and individual difference variables to be specified and uses all available data for each subject, whether complete or partial [35]. Therefore, we included all data in the GEE model. The sample size required for a power of 0.8 was 103 participants, which was calculated using the G-power version 3.1 with α = 0.05, effect size = 0.15 based on Cohen’s recommendation [35]. As we assumed a 35% attrition rate based on extant literature, we recruited 159 community-dwelling older adults. SPSS statistics 25.0 (IBM) was used for analysis. Written informed consent was obtained from the participants. The study was approved by the institutional review board of Chang Gung medical foundation (IRB approval number: 201801317B0).

## Results

The study recruited a total of 159 older adults with an average age of 75.26 years. Most were females and with an elementary school degree. On average, older adults have 1.29 diseases with hypertension and diabetes as the most common ones. Moreover, 25.8% of the participants reported falls in the previous year; the majority (86.8%) maintain exercise habits. We classified the participants into the regular and multicomponent groups through purposive sampling. The study found no significant difference in terms of age, gender, level of education, number of diseases, falls, exercise habits, and frailty between groups (Table 1).

**Table 1:** Sociodemographic characteristics of the participants

	Regular group (*n* = 90)	Multicomponent exercise group (*n* = 69)	Total (*n* = 159)
Age			
Mean ± SD	75.82 ± 7.61	74.52 ± 7.74	75.26 ± 7.67
Gender			
Male	19 (21.1%)	10 (14.5%)	29 (18.2%)
Female	71 (78.9%)	59 (85.5%)	130 (81.8%)
Education			
Elementary school	60 (66.7%)	46 (66.7%)	106 (66.7%)
Junior high school	12 (13.3%)	13 (18.8%)	25 (15.7%)
Senior high school and above	18 (20.0%)	10 (14.5%)	28 (17.6%)
Diseases			
Mean ± SD	1.20 ± 0.96	1.41 ± 1.10	1.29 ± 1.03
Hypertension (Yes)	40 (44.4%)	31 (44.9%)	71 (44.7%)
Diabetes (Yes)	21 (23.3%)	11 (15.9%)	32 (20.1%)
Fall			
No	67 (74.4%)	51 (73.9%)	118 (74.2%)
Yes	23 (25.6%)	18 (26.1%)	41 (25.8%)
Exercise			
No	12 (13.3%)	9 (13.0%)	21 (13.2%)
Yes	78 (86.7%)	60 (87.0%)	138 (86.8%)
Frailty (0–5)			
Mean ± SD	1.11 ± 0.98	0.97 ± 1.16	1.05 ± 1.06
Robust (0)	27 (30)	34 (49.3)*	61 (38.4)
Prefrailty (1–2)	54 (60)	26 (37.7)	80 (50.3)
Frailty (> 3)	9 (10)	9 (13)	18 (11.3)

Statistics are displayed as Mean ± SD for the continuous variables and n (%) for the categorical variables

For the SFT, functional test, and the frailty status of participants at Time 1, the study found that the regular group exhibited better performance than the multicomponent exercise group in the four-stage balance, back scratch, timed up and go, and 2-minute step tests as well as the ADL index, IADL, and the Kihon checklist. Moreover, the study noted nonsignificant differences in baseline values between the regular and multicomponent exercise groups for all variables (*p* > 0.05). After the three-month intervention, we found that the multicomponent exercise group displayed better performance in the 2-minute step test compared with that of the regular group with a significant difference in the two groups (*p* < 0.05). After 6 months, the multicomponent exercise group displayed better performance in terms of the average scores of the regular group in the timed up and go test, 2-minute step test, and MMSE (Table 2).

**Table 2:** Evaluation of the physical performance (senior fitness test), cognition, and frailty between the two groups

Variables (Mean ± SD)	Regular group (*n* = 90)			Multicomponent exercise group (*n* = 69)			ΔTime 2–1	∆Time3–2
	Time 1	Time 2	Time 3	Time 1	Time 2	Time 3	*P* value	*P* value
Four-stage balance test	3.31 ± 0.74	3.36 ± 0.78	3.36 ± 0.87	3.07 ± 0.93	3.29 ± 0.91	3.41 ± 0.72	0.27	0.18
Back scratch test (cm)	−7.24 ± 14.75	− 10.64 ± 13.84	−6.53 ± 14.45	−9.10 ± 13.88	− 11.63 ± 16.32	−11.22 ± 15.50	0.74	0.98
Sit and reach flexibility (cm)	−1.52 ± 10.08	− 3.16 ± 9.88	0.44 ± 7.63	−1.09 ± 8.06	− 2.69 ± 8.91	−1.97 ± 8.69	0	0.37
Timed up and go (s)	10.06 ± 3.73	9.79 ± 3.88	9.59 ± 3.32	10.33 ± 6.18	9.78 ± 5.01	9.35 ± 3.71	0.52	0.76
2-minute step test (times)	82.21 ± 34.96	91.97 ± 30.89	72.75 ± 25.00*^, a^	75.04 ± 31.16	100.02 ± 31.40	86.95 ± 29.37	0.010*	0.42
IADL	0.46 ± 2.03	0.41 ± 1.62*^, a^	0.24 ± 1.33	1.19 ± 3.31	1.75 ± 4.25	1.16 ± 2.69	0.08	0.33
ADL	0.40 ± 1.41	0.35 ± 1.33	0.07 ± 0.31	0.70 ± 2.40	0.83 ± 2.45	0.38 ± 1.28	0.54	0.09
MMSE	0.10 ± 0.43	0.06 ± 0.28	0.15 ± 0.58	0.07 ± 0.31	0.08 ± 0.32	0.03 ± 0.16	0.53	0.10
Kihon	4.79 ± 3.16	5.59 ± 3.79	4.53 ± 2.80	5.09 ± 4.06	6.15 ± 4.34	5.76 ± 3.80	0.53	0.55
QOL	9.68 ± 0.75	9.69 ± 0.84	9.83 ± 0.42	9.41 ± 1.09	9.40 ± 1.27	9.57 ± 1.19	0.88	0.18
Frailty (0–5)	1.11 ± 0.98	0.99 ± 1.11	1.41 ± 0.97	0.97 ± 1.16	0.92 ± 1.16	1.27 ± 0.93	0.52	0.49
Robust (0)	27 (30)	39 (43.3)	11 (12.2)	34 (49.3) *	33 (47.8)	8 (11.6)	–	–
Prefrailty (1–2)	54 (60)	37 (41.1)	41 (45.6)	26 (37.7)	26 (37.7)	25 (36.2)	–	–
Frailty (> 3)	9 (10)	10 (11.1)	7 (7.8)	9 (13)	6 (8.7)	4 (5.8)	–	
Dropout	0	4 (4.4)	31 (34.4)	0	4 (5.8)	32 (46.4)	–	–

Statistics are displayed as Mean ± SD for the continuous variables and n (%) for the categorical variables

* *p* < .05; ** *p* < .01; and *** *p* < .001. ^a^ Significant difference between the regular and multicomponent exercise groups across Times 1–3

For the long-term effect of frailty trajectories, the study proposes that age progression, being females, lack of exercise habits, longer duration in timed up and go, and high scores in the Kihon checklist increase the probability of frailty (*p* < 0.05). Alternatively, more steps in the 2-minute step test and undertaking the multicomponent exercise program of the experiment group reduced the probability of frailty in the long run (*p* < 0.05; Table 3).

**Table 3:** Generalized estimating equation model: multi-exercise intervention and frailty trajectories

Variables		Beta	95% CI	Exp (B)	95% CI	*P* value*
Age		0.02	0.01–0.04	1.02	1.01–1.04	< 0.001***
Gender (Ref: male)	Female	0.23	0.02–0.44	1.26	1.02–1.56	0.03*
Group (Ref: regular group)	Multicomponent exercise group	−0.18	−0.35–0.02	0.83	0.71–0.98	0.03*
Hypertension (Ref: no)	Yes	−0.03	− 0.18–0.13	0.97	0.84–1.14	0.74
Diabetes (Ref: no)	Yes	−0.06	− 0.25–0.13	0.94	0.78–1.14	0.55
Community Care Stations (Ref: no)	Yes	0.33	0.13–0.52	1.39	1.14–1.68	< 0.001***
Fall (Ref: no)	Yes	−0.05	−0.22–0.12	0.95	0.80–1.13	0.55
Exercise (Ref: no)	Yes	−0.94	−1.15–0.72	0.39	0.32–0.49	< 0.001***
Four−stage balance test		−0.03	− 0.14–0.08	0.97	0.87–1.09	0.61
Perceived health status		−0.07	− 0.17–0.04	0.94	0.85–1.04	0.21
Back scratch test (cm)		−0.00	− 0.00–0.00	1.09	0.99–1.00	0.30
Sit and reach flexibility (cm)		−0.01	0.00–0.01	1.00	0.99–1.00	0.28
Timed up and go (s)		0.04	0.02–0.07	1.04	1.02–1.07	0.001**
2 − minute step test (times)		−0.01	0.00–0.01	0.99	0.99–1.00	< 0.001***
IADL		0.00	−0.05–0.05	1.00	0.95–1.05	0.99
ADL		0.04	−0.03–0.12	1.05	0.97–1.12	0.23
MMSE		0.03	−0.18–0.23	1.03	0.84–1.26	0.78
Kihon		0.08	0.05–0.11	1.08	1.05–1.11	< 0.001***
QOL		0.07	−0.05–0.18	1.07	0.96–1.20	0.25

Ref. = reference group. * *p <* .05; ** *p <* .01; and *** *p <* .001

## Discussion

The current study demonstrated that multicomponent exercise could reduce the probability of frailty compared with the regular group. In addition, growing old, being females, lack of exercise, longer duration in the timed up and go, and high scores in the Kihon checklist increase the probability of frailty. Moreover, more steps in the 2-minute step test reduce the probability of frailty.

### Frailty distribution

The study found that 11.3% of the older adults are classified under frail, whereas 50.3% belong to the prefrailty category. This result was consistent with those of other studies. For example, Long-Term Care Plan 2.0 projected an estimated population of older adults with frailty of approximately 5–16% in Taiwan [36]. Other surveys demonstrated that the proportion of older adults with frailty worldwide was 7.4–12.8% [21, 37–40], whereas those at prefrailty ranged from 21 to 48.7% [21, 37–42]. In conclusion, we find that the distribution of frail and prefrail older adults is corresponds to those of other studies.

### Factors associated with social demographics and physical performance with frailty trajectory

The current study found that growing old [40, 42–44], being female [40], and lack of exercise [21, 39] will increase the probability of the frailty trajectory. Regarding physical performance, several cross-sectional studies and systemic reviews demonstrated that physical performance was associated with frailty [23, 25]. Moreover, these studies examined the multicomponent intervention difference using pre- and post-tests [18, 20, 45, 46]. However, only a few studies proved that relation between physical performance of lower extremity ability, such as short physical performance battery (SPPB) or single indicators and frailty in the long run. A study conducted in Brazil examined 353 older adults’ relation between associated factors with frailty over 2 years and found that low scores in the SPPB could predict the high probability of frailty [44]. One longitudinal study on aging in Amsterdam observed the 25 predictors of frailty, such as sociodemographic, lifestyle comorbidity, and physical activity, and found that grip strength, which is an indicator of physical performance, is not associated with frailty [43]. The relation between physical performance and frailty thus required further clarification, and the current study provides evidence that indicators of physical performance, such as the timed up and go and 2-minute step tests within the SFT, could predict frailty in the long run.

### Effect of multicomponent exercise on physical performance and frailty

The findings of the current study are consistent with previous research demonstrating that multicomponent exercise can reverse frailty [8, 16–22]. After an intervention that lasted 12 weeks, the two groups in current study displayed decreased scores for frailty. This notion is especially true for the multicomponent exercise group, whose frailty proportion decreased from 13% (Time 1) to 8.7% (Time 2) to 5.8% (Time 3).

Previous studies reported that cardiorespiratory endurance will decrease 3–6% per year without exercise. By contrast, regular exercise could maintain the cardiorespiratory endurance and function of older adults [8, 17, 44, 47, 48]. This finding corresponds to that of the current study, which found that that a multicomponent exercise program conducted over 12 weeks could improve the indicators of physical performance, especially the 2-minute step test, particularly in the multicomponent group, and increase the cardiorespiratory endurance of older adults. For flexibility, we found no significant difference in the upper and lower extremity tests, such as the sit and reach flexibility and the back scratch test. Previous studies proved that improvement in flexibility may occur across 20–24 weeks with training at two times per week [49]. In addition, the control group displayed a decreased flexibility compared with the intervention group and demonstrated that a lack of exercise could lead to heavy deterioration with frailty compared with the intervention group. The results indicate an improvement in older adults with the exercise intervention and an increased capacity for functional fitness. For the balance test, a meta-analysis reported that the multicomponent exercise program conducted across 12 weeks could improve balance function. This result differed from that of the current study. A possible reason could be that the frequency of sessions in previous studies was more than twice per week [50], whereas the current study conducted only one session per week.

However, maintaining the exercise habit after intervention is recognized as a significant issue in the field of health promotion It is important to help older adults develop and increase their awareness of and skills and abilities in exercise [51]. In addition, a study in the Lancet highlighted a knowledge gap, where physical activity guidelines for older adults have not been fully integrated into primary and geriatric medical practice and are missing from the core training of medical doctors and other healthcare providers [52]. The Taiwanese government has launched several national projects to support greater engagement in exercise in Taiwan, such as programs aiming to prevent or delay disability in the community. This initiative has established certified 266 programs in Taiwanese communities that promote physical activity, nutrition, cognition training, fall prevention, oral health, and complementary therapy [53]. Furthermore, the Department of Health in Taiwan has established groups that monitor and supervise the quality and implementation of community interventions [54]. However, the effects of multicomponent exercise programs are short-lived. Therefore, the Taiwanese government still needs to develop more concrete strategies to assist older adults in maintaining their exercise habits, such as devising feasible policy actions. Moreover, it should increase coordination between government and nongovernment stakeholders, using community consensus and communication to raise awareness of the benefits of physical activity for older adults. Additionally, it should use technology and innovation to create environments conducive to increased physical activity, especially in low-resource areas [55].

## Conclusions

This study is the first to discuss the relation between the indicators of physical performance and frailty trajectory among older adults in Taiwan. Furthermore, we proved that the multicomponent exercise program was effective in improving the frailty status of the participants. However, this study has its limitations. First, the participants were selected through purposive sampling; thus, it probably they may be concerned about their own health and activities in the community. Therefore, external validity may be limited. However, we found no significant difference in all sociodemographic information, physical performance, and frailty between the participants of the current study and those who withdrew before the follow up (*p* > 0.05; Table 4). The current study provided valuable evidence among multicomponent exercise, physical performance in the 2-minute step test, the timed up and go, sociodemographic, and frailty.

**Table 4:** Comparison between the final participants and dropouts from the study

		Final participants (*n* = 96)	Dropout (*n* = 63)
Age	Mean ± SD	74.78 ± 7.32	75.98 ± 8.11
Gender, n (%)	Male	19 (19.8)	10 (15.9)
	Female	77 (80.2)	53 (84.1)
Education, n (%)	Elementary School	65 (65.1)	41 (65.1)
	Junior High School	11 (11.5)	14 (22.2)
	Senior High and above	20 (20.8)	8 (12.7)
Diseases	Mean ± SD	1.24 ± 1.08	1.37 ± 0.94
Fall, n (%)	No	68 (70.8)	50 (79.4)
	Yes	28 (29.2)	13 (20.6)
Exercise, n (%)	No	12 (12.5)	9 (14.3)
	Yes	84 (87.5)	54 (85.7)
Frailty (0–5)	Mean ± SD	0.75 ± 0.65	0.70 ± 0.66
The four-stage balance test	Mean ± SD	3.26 ± 0.85	3.13 ± 0.81
Back scratch test (cm)	Mean ± SD	−7.78 ± 15.01	−8.46 ± 13.42
Sit and reach flexibility (cm)	Mean ± SD	−1.16 ± 8.71	−1.56 ± 10.04
Time up and go (sec)	Mean ± SD	9.93 ± 4.15	10.56 ± 5.94
2-minutes step test (times)	Mean ± SD	83.2 ± 35.44	72.86 ± 29.35
IADL	Mean ± SD	0.48 ± 2.04	1.22 ± 3.4
ADL	Mean ± SD	0.30 ± 1.40	0.87 ± 2.45
MMSE	Mean ± SD	0.07 ± 0.36	0.11 ± 0.41
Kihon	Mean ± SD	4.88 ± 3.48	4.98 ± 3.73
QOL	Mean ± SD	9.63 ± 0.91	9.46 ± 0.93

Statistics are displayed as Mean ± SD for the continuous variables and n (%) for the categorical variables

## Data Availability

The datasets generated and analyzed during the current study are not publicly available because the data were collected through face-to-face interviews but are available from the corresponding author and participants on reasonable request.

## Abbreviations

ADL: Activities of daily living
IADL: Instrumental activities of daily living
MMSE: Mini-mental state examination
QOL: Quality of life
SOF: Study of osteoporotic fractures
SFT: Senior fitness test
GEE: Generalized estimating equation
SPPB: Short physical performance battery
